# Quantitative multiplex immunohistochemistry with colorimetric staining (QUIVER) may still benefit from MILAN

**DOI:** 10.1186/s40478-023-01585-y

**Published:** 2023-06-07

**Authors:** Maddalena M. Bolognesi, Asier Antoranz, Francesca Maria Bosisio, Giorgio Cattoretti

**Affiliations:** 1 Pathology, Department of Medicine and Surgery, Università di Milano-Bicocca, Via Cadore 48, Monza, MI Italy; 2 Translational Cell and Tissue Research Unit, Department of Imaging and Pathology, KU Leuven, Leuven, Belgium; 3 The Laboratory for Precision Cancer Medicine, Translational Cell and Tissue Research Unit, Department of Imaging and Pathology, KU Leuven, Leuven, Belgium

**Keywords:** Multiplex staining, Neuropathology

Dear Editor,

We read with interest the publication by Ryan K. Shahidehpour, et al. The localization of molecularly distinct microglia populations to Alzheimer’s disease pathologies using QUIVER. Acta Neuropathol Commun 11, 45 (2023) [1].

Cyclic multiplex immunostaining consists of cycles of staining, imaging, removal of the signal and repetition with another set of antibodies. Removal of the primary and secondary antibodies [2], quenching [3, 4] or removal [5, 6] of the fluorochrome for directly-conjugated primary Abs or removal of the pigment deposited via immunoperoxidase [1, 7], are all methods to accomplish the task (see [8] for a comprehensive review). Glass et al. [9] published first the latter method in 2009 on human and mouse routinely fixed (FFPE) brain.

The physical and chemical energy required to remove the signal differ in each of the above methods and affects both the tissue antigens and the signal-bearing molecule.

Selective enzymatic removal of the antibody [5] or fluorochrome [6] is very effective and minimally affects the tissue. Quenching a labile fluorochrome [3] is also effective and tissue-sparing; not so is quenching a fluorochrome designed for stability under excitation [4]. In this case, UV light exposure and high pH, required to exhaust the fluorochrome, causes progressive tissue loss [10].

The removal of a light-absorbing pigment, as with Shahidehpour, et al., requires: (A) a removable substance such as the alcohol-soluble AEC (aminoethyl carbazole) light microscopy pigment, (B) transferring the stained section to ethanol or xylene [7] and back to buffer. After the removal of the AEC precipitate, the exogenous peroxidase from the previous immune cycle is inactivated with a methanol + hydrogen peroxide mixture.

The task of antibody removal in this technique is delegated to the exposure to high-heat in a buffer (the antigen retrieval solution, AR), a very inconsistent stripping method [11]. Although AR may re-expose epitopes re-masked during the ethanol/xylene passages [2, 12], it is deleterious to tissue antigens in the cycling protocol used by Shahidehpour, et al. [13].

We developed the MILAN technique [2, 14], in which bound primary and fluorochrome-conjugated secondary antibodies are efficiently removed, as published by several groups [15–19], including FFPE human brain in 2020 [20, 21].

In MILAN, a combination of a reducing agent (ßMercaptoethanol) and a detergent (SDS) disassembles and removes the bound antibody(ies).

Tissue antigenic epitopes are preserved: after more than 30 cycles of staining and stripping the signal intensity variations are less than 10% for six of eight antibodies tested (the other two increased the signal) (see Suppl; Fig. 1 in [16] and [22]). This has been demonstrated by quadruplicate 10-cycle experiments [2], compared to a single cycle in the Shahidehpour, et al. manuscript.

Removal of antibodies, primary and secondary, bound to the tissue, turned out to be quite more difficult than gleaning from existing published data because of three reasons: (A) affinity for the tissue epitope [11], (B) intrinsic resilience of the 4-chain immunoglobulin complex [2] and (C) propensity of the bound antibodies to denaturation and in-situ precipitation because of loss of protein-associated water during cycling [23]. In situ denatured antibodies cannot be removed but can still be detected [23].

The two antibody-removal buffers we published [2] can efficiently remove bound antibodies if preservation of both the tissue antigens and the antibody native conformation is obtained by adding disaccharides in all the staining cycle steps [2, 23], including slide mounting.

Shahidehpour, et al. should be credited for attempting to adapt the MILAN technique to light microscopy via the deposition of an alcohol-soluble pigment, however their adaptation may have caused in multiple steps the troubles they describe (i.e. failure to remove bound antibodies with MILAN).

The most damaging, but obligate, step introduced by Shahidehpour, et al. in their modified method is a 70–100% Ethanol (a protein coagulant) and a very low pH Hydrochloric acid bath (a denaturant; this latter, a step not previously reported in this field). The effect of the ethanol-HCl mixture is to coagulate and precipitate bound antibodies, rendering these resistant to any removal buffer, including MILAN, but still recognizable by polyclonal secondary antibodies.

In addition, no disaccharide is added to the buffers [23], the mounting medium is of unknown effect on antigens and antibodies during cycling [2], sections are washed in running tap water, an antigen masker [24].

With hindsight, performing the antibody stripping step with MILAN before removing the chromogen, may have solved the problem and avoided the complex problems described for the alternative procedure chosen.

We agree with Shahidehpour, et al. that entirely avoiding autofluorescence is one of the solutions. However, the throughput feasible via multiple simultaneous antibody deposition is a clear advantage of MILAN and other similar methods. Autofluorescence can be avoided by the use of fluorochromes [25] and light sources away from the most obnoxious wavelengths.

## Data Availability

Not applicable.
